# Employability in health professional education: a scoping review

**DOI:** 10.1186/s12909-022-03913-7

**Published:** 2023-01-17

**Authors:** Delyse Leadbeatter, Shanika Nanayakkara, Xiaoyan Zhou, Jinlong Gao

**Affiliations:** The University of Sydney School of Dentistry, Sydney Dental Hospital, 2-18 Chalmers Street, Surry Hills, Sydney, NSW 2010 Australia

**Keywords:** Employability, Health professional education, Graduate outcome

## Abstract

**Background:**

The concept of employability can help educators understand the variability in the career outcome of graduates. Within the health professional education (HPE) literature, various conceptions of employability are used and implied. This review considers how the concept ‘employability’ is depicted and characterised in HPE literature.

**Methods:**

A scoping review was conducted. The authors searched Medline, Web of Science and Scopus databases for English language literature relevant to employability in HPE. Arksey and O'Malley’s review protocol and the criteria defined in the preferred Reporting Items for Systematic Reviews and Meta-Analysis Extension for Scoping Reviews Checklist (PRISMA-ScR) were used with methodological guidance provided by Levac et al. and Peters et al. with the exception of formal stakeholder consultation.

**Results:**

The search resulted in 158 articles, of which 34 articles were included in this review. Charting the included articles revealed that within the set of articles, there is much diversity in study design, geographical setting and health profession. Three conceptions of employability were identified: acquiring a professional job, sustaining employment and thriving in the workforce.

**Conclusion:**

Conceptions of employability in HPE are largely focused on listing skills and capabilities for entry into employment and sustaining a career. To address gaps in research, structural contributions to employability and institutional strategies to promote conditions for thriving in disruption should be explored.

## Introduction

The idea of employability has been used to describe the success of obtaining and maintaining graduate jobs in labour markets [1], however there is no single, shared definition or conception of graduate employability. Hora et al. [2] traced the history of employability as first used to describe training initiatives for people with disabilities and disenfranchised workers and Bridgstock and Jackson [3] claim that employability began to be associated with the production of human capital through university education since the 1990s. Employability has come to firmly represent generic and discipline-specific knowledge, skills and attitudes of graduates [4]. Critique of the individual skills-focused orientation of employability has resulted in attention being paid to other approaches to understanding the complexity of graduate employability.

Holmes [5] critiques the characterisation of employability as the possession of lists of skills and capabilities as bearing little relation to employment outcomes, and that students with similar skills can have different outcomes. He suggests that there is more to employability than attending to human capital, by identifying position, or social capital and process, or career self-management as key aspects of employability. Similarly, Behle’s holistic framework of employability that takes into account both individual factors and structures impacting graduate employability [6], aligned with what Holmes [5] refers to as the influence of social positioning on employability. Behle [6] argues the effect of gender, ethnicity, social class, age, health status or nationality should be accounted for and that labour market factors are influential. To organise the various dynamics and influences, Tomlinson describes three levels through which employability can be viewed, including the macro of economies and labour markets, the meso of university and workplace, and the micro of an individual’s psycho-social and biographical development [7]. Critical perspectives on the concept of employability demonstrate that employability discourses are not neutral or benign representations of individuals’ self-management in the labour market [8, 9].

Since the 1990s, research about graduate employability in higher education has (HE) kept advancing theoretically, however, it has not been as popular in health professional education (HPE). Several reasons have been proposed to explain why engagement with employability has been limited in HPE. The focus on achieving professional competencies, as defined by regulators, can project a perception that employability is addressed by university programs concentrating on the key skills needed by graduates [10]. Conflation of the terms: employment and employability, along with heterogeneity of employers across health disciplines has contributed to limited scholarship about graduate employability in HPE [11]. Another explanation is that scholarly works contributing to employability research in HPE can be embedded in different disciplines and communities, making them less visible. For example, Healy et al. [12] note the relevance to employability of scholarly works about professional identities in HPE and biomedical career development.

As identified by Bell et al. [13], while all graduates have been deemed to have met threshold standards specific to their discipline, some graduates go on to be more employable than others and succeed in their careers in complex and unpredictable environments. The health sector has been less affected than others by fluctuations in the labour market until more recently. Health professionals nowadays are more likely to have varied career pathways than a single-professional track. Throughout their life, graduates could take on jobs that are different to that of their initial profession [14]. Thus, the notion of job security in a changing labour market landscape provides a stimulus to explore how health profession graduates navigate their careers. The COVID-19 pandemic and post-pandemic state has posed challenges to healthcare systems and conventional practices. Workforces are required to be prepared for, in-tune with, and quickly adapt to changing priorities and healthcare models. Graduates, including health professionals, are expected to recognise the need for conviviality for a new normality and thriving can emerge with proliferating challenges and dramatic change in complex and turbulent environments [15, 16]. Thus, the issue that arises for understandings of employability as a concept for HPE goes beyond equipping students with the requisite knowledge, skills and behaviours for their chosen profession, but one of considering other aspects that supporting career development and conditions to thrive in context. Römgens et al. [17] synthesis of dimensions of employability provides some guidance about the importance of flexibility and adaptability, social recognition and situating work in the broader context of life. To take the importance of sense of self, meaningful work and societal contribution further, we adopted thrivability as a theoretical perspective to capture these aspects in the HPE employability literature.

Today’s workforces comprise of a range of interdependent stakeholders working together. They are influenced by multiple internal and external factors and networked to form a hyperconnected workforce ecosystem [18]. Thrivability is the persistent intention and ability to be generative and flourish in changing and unsettling environments, and to create more value than we consume [15]. Thriving is said to emerge with proliferating challenges and dramatic changes in complex and turbulent environments. Thus, it could be argued that thrivability is increasingly relevant in a COVID and post-COVID shaped health care system and shared global concerns such as environmental and economic crises. According to Laszalo [16], there are four interdependent coherence domains for human thrivability: personal, interpersonal, transpersonal, and temporal domains that focus on people in their environments. Strengths that promote thriving include openness, conviviality, resilience, agility, and diversity. As such, thrivability recognises that the typical capabilities associated with employability may not be sufficient when it comes to preparing students for work and takes further Römgens et al.’s dimensions of flexibility and individual identities within organisation’s goals and values [17]. Thrivability is aligned with employability as a psychosocial process that promotes personal and career adaptability within the context of labour market challenges and opportunities [19, 20].

### Purpose and research question

This scoping review considers the key conceptions of employability in published HPE literature to explore how the concept of employability could illuminate reasons that make some graduates more or less employable than others. The central question guiding this review is: How is the concept ‘employability’ used in HPE literature?

## Methods

A scoping review was selected as the methodology to map out an area of study that has not been reviewed comprehensively [21] with the aim of informing future education programs, practice and research [22]. We followed the criteria defined in the preferred Reporting Items for Systematic Reviews and Meta-Analysis Extension for Scoping Reviews Checklist (PRISMA-ScR) [23], the methodological guidance provided by Levac et al. [24] and Peters et al. [25], with the exception of formal stakeholder consultation, when developing this scoping review. The review comprised five stages: 1) defining the research question; 2) identifying relevant studies; 3) selecting the studies; 4) charting the data; and 5) collating, summarizing, and reporting the results. The purpose and research question outlined above fulfil the first stage and descriptions of the remaining four stages follow.

### Identifying relevant studies

**Fig. 1:**
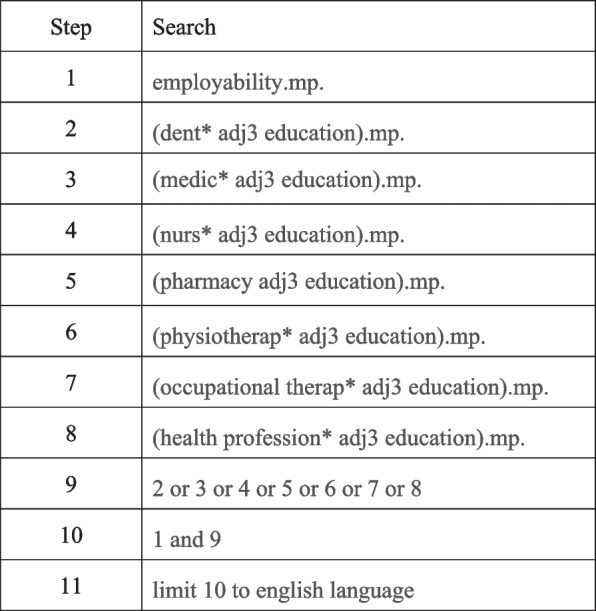
Search strategy- Medline via Ovid

We consulted the faculty liaison librarian and used the following search terms for the database search: employability, dental education, medical education, nursing education, pharmacy education, physiotherapy education, occupational therapy education, veterinarian education and health professional education. While veterinarian education is not typically clustered with HPE, we included it in the search because the nature of practice is similar to health practice. We searched through the following three online databases: Medline via Ovid, Web of Science and Scopus and limited the articles to English language. The first systematic search was conducted on 20^th^ July 2020 and the follow up search was on 4^th^ February 2021 to capture any new publications. Figure 1 shows the full search strategy used in Medline via Ovid. Selected articles were managed using EndNote X9 (Clarivate Analytics, Philadelphia, United States) and duplicates were removed. A grey literature search was also conducted to capture any additional articles or resources available.

### Study selection

**Fig. 2:**
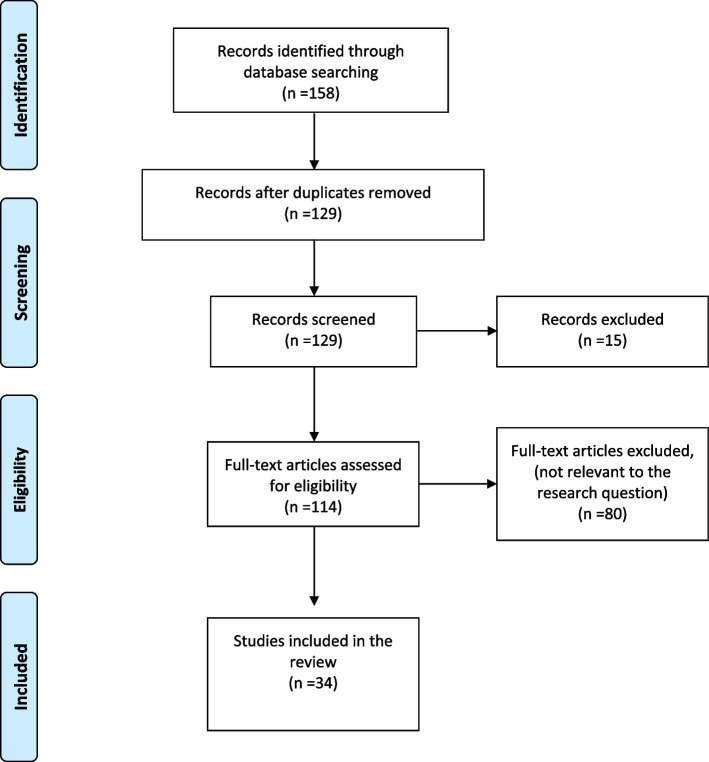
PRISMA-ScR flow diagram of study selection process

The search resulted in 158 articles (54 in Medline, 30 in Web of Science and 73 in Scopus, 1 in grey literature search). After removing the duplicates (*n* = 29), 129 articles were selected for screening. Articles that were not in English language (*n* = 3) and the articles without access to full text (*n* = 12) were excluded and 114 articles were selected for second stage screening. Eighty articles were excluded during this process as they were not relevant to the research questions of this review. The authors selected 34 articles for in-depth reading and data extraction. The Prisma-Scr flow diagram (Fig. 2) outlines the article selection process.

The first level screening for relevance by titles, abstracts and keywords was undertaken independently by all researchers based on the inclusion and exclusion criteria described below. Second level screening involved reading of full text papers for their relevance to the research questions. Researchers were assigned papers evenly and a second researcher was assigned to papers that were initially classified to be excluded or ‘not sure’. To facilitate this screening process, a shared sheet was set up in Microsoft Teams and papers were discussed before taking a decision to exclude articles from the review. Three meetings provided a way to reach consensus on the final set of 34 included papers. Reasons for inclusion and exclusion were recorded for each paper.

Inclusion criteria included publications in English that mentioned the concept of employability as an educational goal or as a graduate outcome of an entry-level health professional education program. Research design was not limited; thus, empirical studies, conceptual papers and commentaries were included. Studies included the perspective of education providers, students, employers and consumers. Articles were not limited by the date of publication.

Exclusion criteria comprised publications where full text was unavailable, articles not in English and those which focused on post-graduation training programs or continuing professional development courses. Papers that focused on program and course evaluations, were excluded, along with papers researching the employability of specific groups (e.g., academics and people with health conditions).

### Charting the data

A data charting form was shared among the researchers using Microsoft Teams. Data extracted included: article identifiers, geographical location, health profession, study design and perspective of the research. A team meeting was held to ensure a shared mental model for charting of data. Quality appraisal was not conducted to capture the breadth of available literature as these are not common in scoping reviews [21].

### Collating, summarising and analysing the data

An initial overview of the type and distribution of literature about employability in HPE was conducted by systematically counting the geographical distribution of publications, health profession of interest, year of publication and methodology employed in the papers. A table of article characteristics and findings was generated to catalogue the included articles. Descriptive statistics were calculated using Microsoft Excel program.

To analyse the conceptions utilised in the included papers about employability in HPE, we conducted a thematic analysis [26] to map the use of employability concepts in HPE. The theoretical  perspective that was used within the analysis included Russell’s Thrivability [15]. Members of the research team independently conducted in-depth reading and initial mapping of the key conceptions presented in the papers before meeting to share, discuss and finalise conceptions of employability in HPE.

## Results

### Descriptive summary

**Fig. 3:**
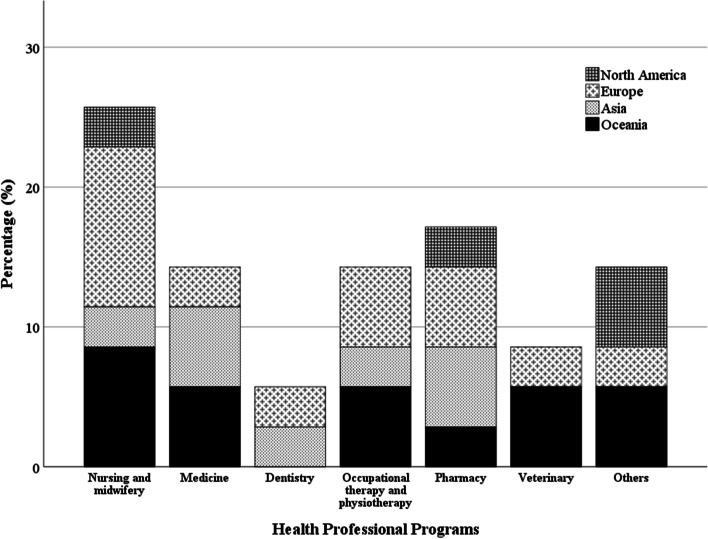
Study distribution based on health professional program and region

Geographical setting of the included articles was diverse. Of the included publications that originated from Europe (*n* = 12, 35.3%), half originated from the United Kingdom (*n* = 6). There were twelve articles from Australia (35.3%) while seven were from Asian countries (20.6%) and four originated from United States (11.8%). Cross-sectional studies utilizing surveys or focus group interviews constituted most of the publications (*n* = 20, 58.8%). Considering the health profession, 26.5% of the publications were based on nursing and midwifery education. Pharmacy, occupational therapy and physiotherapy, medical, dental and veterinary education programs contributed 17.6, 14.7, 14.7, 5.9, and 5.9% respectively. Figure 3 shows the study distribution based on health professional program and region. Table 1 summarises details of the selected articles.

**Table 1:** Summary of the selected articles

First author and year of publication	Study Design/ Article type	Subjects/Program	Country/region	Perspectives
Ang H et al. (2013) [27]	Cross sectional-survey (quantitative)	Pharmacists	Singapore	Employee
Bell MA et al. (2018) [13]	Scoping review	Veterinary practice	Australia	Stakeholders (Employer/employee/team/client/profession)
Bennett D et al. (2020) [28]	Cross sectional-survey (mixed methods)	Medical and health-related disciplines, engineering	Australia	Student
Biesma RG et al. (2008) [29]	Cross sectional-survey (quantitative)	Public health	United Kingdom, Netherlands and Poland	Employer
Brewer M et al. (2014) [30]	Cross sectional-survey (quantitative)	Health Sciences	Australia	Student
Brick NK and Bolton L (2013) [31]	Commentary	Nursing	United Kingdom	Student
Cake MA et al. (2016) [32]	Systematic review	Veterinary practice	Australia, United Kingdom	Student, veterinary graduate, veterinarian, employer, academic, client (pet owners), professional or industry bodies
Cake MA et al. (2022) [11]	Model Development	Veterinary practice	Australia, United Kingdom	Employee, employer, academics, industry
Donik B et al. (2015) [33]	Cross sectional-survey (quantitative)	Nursing	Slovenia	Student, employer
Falk AEL et al. (2012) [34]	Project evaluation	Occupational therapy, physiotherapy, social work, nursing, public health	Europe	Student, education provider, EU commission
Gerrard S and Billington J (2014) [35]	Cross sectional-focus group (qualitative)	Nursing	England	Student
Hanks S et al. (2018) [36]	Cross sectional-survey (mixed methods)	Dentistry	England and Wales	Employer
Harolds JA et al. (2013) [37]	Cross sectional-survey (quantitative)	Nuclear medicine	United States	Student (resident)
Hora MT et al. (2019) [38]	Cross sectional-survey (mixed methods)	Nursing and engineering	United States	Student, education provider, employer
Hughes I (2002) [39]	Commentary	Pharmacology	United Kingdom	Employee (graduates)
Jackson DA and Edgar S (2019) [40]	Cross sectional-survey and focus group (Mixed method)	Physiotherapy and business	Australia	Student, career advisor
Jacob ER et al. (2016) [41]	Cross sectional-survey (qualitative)	Nursing	Australia	Education provider
Lawton V et al. (2021) [42]	Longitudinal observational study	Physiotherapy	Australia	Student
Li G (2017) [43]	Quantitative curriculum evaluation	Medicine, law, science and arts	China	Education provider
Marshall JE et al. (2017) [44]	Qualitative (longitudinal phenomenological approach)	Midwifery	United Kingdom	Student
Nash RE et al. (2015) [45]	Systematic review	Pharmacy	Australia	Education provider
Nguyen NB et al. (2020) [46]	Scoping review	Nursing	Australia	Employer, employee, team, client, profession
Nielsen CJ et al. (2020) [47]	Cross sectional-survey (quantitative)	Nuclear medicine	United States	Employee
Ramil A et al. (2010) [48]	Cross sectional-survey (quantitative)	Physiotherapy	Malaysia	Employer
Ross et al. (2014) [49]	Cross sectional-survey (mixed methods)	Medicine	Europe	Student, education provider, employee, employer, patient
Satwik (2016) [50]	Cross sectional-survey (quantitative)	Dentistry	India	Student
Singh S (2016) [51]	Commentary	Pharmacy	India	Education provider
Steeb D et al. (2021) [52]	Cross sectional-survey (mixed methods)	Pharmacy	United States	Student
Sutherland K and Ho S (2017) [53]	Cross sectional-survey (mixed methods)	Medicine, law, science and arts	Australia	Student
Tavares O (2017) [54]	Cross sectional-survey (qualitative)	Pharmacy Arts and Engineering	Portugal	Student
Theobald K et al. (2021) [55]	Co-design methodology	Nursing	Australia	Student
Thew M et al. (2018) [56]	Cross sectional-survey and focus group (Mixed method)	Occupational therapy	United Kingdom	Employee
Wu L et al. (2014) [57]	Commentary	Medicine	China	Education provider
Ye et al. (2014) [58]	Cross sectional-survey (quantitative)	Nursing	China	Employee and employer

### Three conceptions of employability in HPE

#### Employability in HPE is acquiring a professional job

Employability was widely perceived as professional job acquisition by graduates from professional degrees in many of the included articles  [27, 29, 32, 37, 41, 47, 49, 53, 56, 57]. This conception focuses on employability as the skills and expertise required to acquire a professional job. Embedded in this conception is both the universities’ understanding of the employment context along with employer and industry expectations [43, 48, 51, 54]. Another facet of this conception is that acquiring a job is influenced by hard-to-control external factors such as rules and regulations of the labour market [34, 54].

Disciplinary expertise and procedural competence were considered essential to acquiring a professional job [11, 30, 31, 33, 36, 39, 41, 45–48, 50, 57, 58]. ‘Non-technical’ skills were also identified as key graduate requirements [31, 32, 40]. Thus, individual abilities in effective communication [11, 13, 30, 32, 35, 36, 38, 42, 58], teamwork [29, 30, 58], social media proficiency [53] and time management [35], were associated with the ability to acquire a professional job. Included articles researching employer perspectives found that employers seek graduates with skills and characteristics similar to those identified above [11, 13, 43, 48, 53].

Researchers recognised that HE institutions play a major role in helping students develop the necessary skills to transition from university to the working environment [28, 35, 42, 44, 53, 56, 59, 60]. Specific activities were shown to prepare graduates for successful transition [27, 35, 37, 39, 47, 56]. For example, speciality and residency training programs along with clinical and outreach placements in university curricula were valued for enhancing employability [37, 42]. In particular, variation in clinical placement sites and the number of clinical hours was associated with increased chance of acquiring a professional job [42, 47]. Support with job applications and interviews, such as mock interviews, assisted with job acquisition [31]. Skills gained through life experience and work experience in extra-curricular activities were seen as beneficial for employability, for example, part-time or vacation employment, involvement in student representative committee, and a magazine editorial team [35, 39].

#### Employability in HPE is sustaining employment

This conception focuses on employability as having a temporal dimension and included articles identified factors needed to sustain employment and progress a career [11, 13, 32, 54, 59, 60]. Integral to this conception is the idea that a successful career relates to individual satisfaction [11, 13]. This conception begins to acknowledge that employability, while predominantly concentrated on the attributes and achievements of individual employees, positions them firmly in the workforce, community, and the economy, thus echoing Knight and Yorke’s [61] view of employability [35, 38, 48, 55].

According to several included articles, graduates should have attributes desired and valued by employers and industry to sustain employment [11, 32, 43, 48, 54]. To be successful and satisfied, a health profession graduate needs to flexibly adapt existing credentials to choose and secure roles [54], and “move self-sufficiently within the labour market” [44]. Employers highly value self-directed learning, autonomous practice, and a willingness to learn and update knowledge over the career [56]. Health practitioners perform multi-faceted work and need to be able to balance multiple stakeholders’ expectations [11, 13]. Thus, many of the articles explored dispositions and capabilities needed for health professionals to be successful and sustainable in employment. While there is overlap with what is needed for securing employment, these focus more on sustainable practices. A capability set put forward by Bell et al. for veterinary graduates include positive personal qualities, self-belief, metacognition, and experience [13]. Self-reflection was named specifically as a practice important to employability [29, 30, 36, 45], along with conflict resolution skills [30]. Further research by Cake et al. identified that a reflective identity is central to employability and that effective practice, productive relationships, professional commitment, and psychological resources all contribute to the formation of a reflective identity [11]. Resilience [32] and self-confidence were associated with employability [13, 33, 58], while self-confidence should be balanced with an awareness of limitations [32, 48].

The role that HE institutions play in helping students and graduates to sustain employment throughout their professional careers was noted. When industry and university collaborated to design nursing programs, the mutually agreed principles were seen to promote the employability of graduates [55]. Continuing professional education (CPE) offered by HE institutions were recognised for their impact on professional practice. A study by Ang et al. reported that mandatory CPE is valued because of the positive impact on professional growth of the pharmacists by enhancing the standards of practice [27].

#### Employability is thriving in the workforce

This conception identifies employability as conditions for individuals to not only acquire and sustain work but to be creative, lead and shape. Integral to this conception is that thriving requires conditions for openness, resilience, agility, and expressions of diversity. Legacy and impact are central to thriving, thus the focus is both on the individual and on the individual in cooperation with others.

The value an individual brings to a workplace relates to the influence they have. Some included articles argued that employability in HPE is not limited to the chosen professional career but refers to the open labour market, and ultimately to realise unbounded potential in the workforce [13, 48, 54]. Mason et al. and Donik et al. claimed that employability refers to “work willingness”, such that “a health professional will creatively contribute to the goals and values of an organisation” [33, 62]. Ye and Jiang took employability further, linking it to identity and a sense of self-worth in society [58]. Client-centredness and relationship-based care [32] were seen as a key aspect of self-awareness about professional identity [30].

Leadership qualities and management skills were identified from the included articles to contribute to thriving of individuals in workplaces and society. Practice and people management skills [30, 36] were recognised to enhance employability. Leadership qualities such as business and entrepreneurial skills [13, 43], networking skills, influence, and creativity [39, 56] enable graduates to navigate contemporary work and guide a team to achieve their vision of success [33].

The interplay between work and life is included in this conception. Students' perceptions of their capacities to work effectively with others is related to quality of life and measures of well-being [63]. The impossibility of separating work and life means that employability should capture the complex combination of living, learning and work [64].

Activities undertaken by health professions students contributed to thriving. Occupational therapy students who undertook ‘non-traditional’ placements were found to be entrepreneurial and occupation-focussed because they were involved in service development and clinical governance during their degree [55]. Pharmacy students who engaged in self-directed placements were able to reflect on values and aspects of jobs that were important and not important to them [52]. Education about issues related to gender, age, disability, and ethnicity was recommended to enhance employability among future health care professionals [34].

## Discussion

This scoping review investigated different conceptions of employability used in HPE literature. The majority of the articles included in this review were published in the last ten years, even though the concept of employability has been explored in HE for over three decades [65]. This signifies increasing interest about employability in HPE. Differences in engagement across the health professions were noted with nursing and midwifery contributing most. In contrast, the concept of employability has been less explored in medicine and dentistry. Limited availability of longitudinal studies to trace career progress of graduates constrains the understanding of the temporal scale of employability. Three distinct yet connected conceptions of employability identified in this study were acquiring a professional job, sustaining employment, and thriving in complex working environments.

Employability was frequently recognised as the ability to acquire a professional job and many papers focused on investigating the knowledge, skills and attributes required for a professional job. Relating employability with employment and the focus on competencies demonstrates that among HPE researchers, employability scholarship is in the early stages. Bridgstock and Jackson [3] argue that concentrating on graduate employment as a measure of employability can inhibit personalised approaches to incorporate graduates’ own goals and aspirations and fail to recognise the different ways that graduates can contribute to society through their work.

The skills and capabilities for sustaining professional practice in the health sector also dominated the theme of employability as sustaining employment. However, the skills already identified to sustain employment in a health professional career may not be sufficient to prepare graduates for the future turbulent and unpredictable labour market such as organisational restructuring, global crises, declining jobs and outsourcing, and emerging and disappearing occupations. In order for individuals to manage their own career in response to these changing patterns and uncertainty, Bates et al. suggested employability is determined by a professional purpose mindset which is a flexible and changeable mindset rather than a fixed disposition [66]. The professional purpose mindset reflects a person's commitment to a professional future founded not only on their personal values and professional goals, but also on social citizenship. Sustaining employment in a challenging and fluctuating working environment is influenced by job characteristics, relational factors, organisational context, and personal factors [5, 6]. Geographical and occupational demand, regulations, such as visa regulations, and the supply of graduates situate the concept of employability firmly beyond the possession of individual capabilities [34, 54, 67]. In defence of the skills-orientation, Pegg and colleagues [67] put forward that employability is not about lists or categories of skills, but “skilful practices in context”, which take into account personal development and career planning. Thus, skilful practices for work engagement and organizational commitment are desired for the graduates to sustain employment in the labour market.

Approaching employability through the lens of thrivability is constructive because of the beneficial outcomes it can bring to the workforce ecosystem [18]. Thrivability recognises that the capabilities-approach associated with employability may not be sufficient when it comes to preparing students for work and understanding the career trajectories of graduates. Considering contribution to the organisation’s values, impact and legacy may offer more productive portrayals of employability which attend to the macro, meso and micro scales introduced by Tomlinson [7]. The concept of thrivability can be more holistically understood by acknowledging the varying perspectives of workforce ecosystem in HPE [18]. Health professions graduates need to meet the expectations of different stakeholders, which interact at multiple societal, organizational and personal levels [11, 13]. Thus, expectations from clients (individuals, families, and communities) must be considered in health professional employability. Society has a great interest in the employability of HPE graduates through the social contract [68], and consumer perspectives are increasingly sought and taken notice of by regulators and education providers [69, 70].

Career development and management is an aspect of the findings of this scoping review worth highlighting. Healy et al. [12] showed that the graduate employability and career development literatures are quite distinct, however offer synergistic potential for the advancement of understanding career success and graduate outcomes. The career ecosystem [71] was used by Donald et al. [72] to articulate the multifaceted interdependencies and interactions that occur at macro, meso and micro levels for graduate careers to unfold, showing the complexities of employability as more than a dichotomous relationship between an employee with certain skills and employer who seeks a skilled worker. Importantly, by recognising that career decisions are integral to employability [73], the common view that employability is concerned with student professionals’ journeys after graduation can be challenged.

### Implications

The opportunities for research about graduate employability and HPE stem from the limited body of literature and the major focus so far, on individual skills and capabilities. We recommend future empirical research about employability to re-balance the current skills and capability focus. Further understanding of the structural and organisational factors influencing and fostering thrivability of employees is needed. Broadening from employee and employer perspectives to include wider stakeholder views of the workforce ecosystem [18] can provide a basis for further research. For instance, employability could provide a mechanism to advance knowledge about collaborations among students and consumers to research what curricular strategies and institutional policies can facilitate the development of thriving professionals. This scoping review found that most studies used cross-sectional designs or focus groups when researching employability in HPE. Methodologically, longitudinal study designs and studies which explore interactions in the complex system or take a dispositional view of employability as a psychosocial process [20] are recommended in the future to strengthen research on employability in HPE.

### Limitations

We conducted a scoping review instead of a systematic review to answer our research questions because our purpose was to identify the extent of, and gaps in knowledge on this topic and to clarify the concepts in order to design future investigations [74]. Although we aimed to be as thorough as possible, our search was limited to three databases and grey literature and for articles in English language. Additional databases may have yielded more articles relevant to this topic and we may have missed information on non-English articles inadvertently. With the aim to explore the nature and extent of the information available in scholarly publications and considering the heterogeneity of the included articles, we did not critically appraise the articles for their quality. However, in-depth analysis and discussion about the content of the included articles helped us to methodically extract the relevant information and synthesise the results of this review.

## Conclusion

In conclusion, the review highlights that employability is gaining more interest in HPE, particularly in the professions of nursing and pharmacy but less so in dentistry and medicine. The conception of employability as synonymous with employment, or acquiring a professional job is widespread in the selected literature, however employability as sustaining employment and thriving in the working environment are well recognised. This interest in employability presents an opportunity for research to shift from a focus on skills and capabilities of graduates to explore how universities can admit and prepare graduates for future labour market changes and turbulent conditions. Furthermore, the findings of this review could provide a foundation from which further research and scholarship on employability in HPE can arise, particularly exploring structural factors, such as social and economic situation, policy and organisational environment, that influence the employability of health professional graduates.

## Data Availability

All data generated or analysed during this study are included in this published article.
